# Global Trends in Research of Treatment on Bladder Cancer with Chinese Medicine Monomer from 2000 to 2021: A Bibliometric Analysis

**DOI:** 10.1155/2022/3382360

**Published:** 2022-09-19

**Authors:** Desheng Li, Minfang Zuo, Xinming Hu

**Affiliations:** ^1^Department of Organ Transplantation, The Second Affiliated Hospital of Hainan Medical University, Haikou 571000, China; ^2^Department of Urology, The Second Affiliated Hospital of Hainan Medical University, Haikou 571000, China; ^3^Department of Chinese Traditional Medicine, The Second Affiliated Hospital of Hainan Medical University, Haikou 571000, China

## Abstract

Bladder cancer is a malignant tumor that occurs on the mucous membrane of the bladder. It is the most common malignant tumor of the urinary system and one of the top ten common tumors in the whole body. This bibliometric analysis was applied to identify the characteristics of global scientific output, the hotspots, and frontiers about treatment on bladder cancer with Chinese medicine monomer over the past 22 years. We retrieved publications published from 2000 to 2021 and their recorded information from Web of Science Core Collection (WoSCC). VOSviewer and CiteSpace were used to analyze bibliometric indicators and visualize the trend and hotspots of researches on bladder cancer with Chinese medicine monomer. Altogether, 658 original articles were reviewed, and the results showed that the annual number of publications (Np) shows an upward trend over the past 22 years as a whole. The US produced the most papers, and the number of citations (Nc) and *H*-index of the US ranked first. Johns Hopkins University and BJU International were the most prolific affiliation and journal, respectively. Recently, the keywords “NF-kappa B” appeared frequently. Besides, quercetin is the most thorough research in the treatment of bladder cancer with Chinese herbal compound, but whether quercetin is the most potent compound needs further study.

## 1. Introduction

Bladder cancer is the most common and high incidence rate malignant tumor in urinary system [1], and the full name is bladder urothelial cell carcinoma, which mainly occurs in the bladder mucosa [2]. The prevalence of male is higher than that of female, and the incidence rate is increasing year by year, which has a serious impact on patients' health [3]. Bladder cancer is characterized by metastasis and recurrence [4]; however, surgery and chemotherapy-based therapies are difficult to improve the metastasis or recurrence of bladder cancer [5]. Moreover, the serious adverse reactions of surgery and chemotherapy have a great impact on the quality of life and prognosis of patients. Therefore, it is very important to find and develop new methods and drugs for the effective treatment of needle bladder muscle cancer. Medicinal plant resources are very abundant all over the world, and a large number of studies have shown that traditional Chinese medicine monomer or extract has good antitumor activity. In addition, it has many advantages such as high selectivity, small body damage, and low toxic and side effects, which has attracted the attention of more and more researchers [6]. For instance, Chen et al. confirmed that isoquercitrin inhibits the progression of bladder cancer in vivo, and the molecular mechanism of this inhibition may be closely related to PI3K/Akt and PKC signaling pathway [7]. Yan et al. demonstrated that berberine may inhibit the metastasis and invasion of bladder cancer cells by blocking the expression of heparinase, so it can be used to reduce the recurrence of bladder cancer in clinic [8]. Li et al. showed that fluoxetine is a new, effective, and safe drug for bladder chemotherapy [9]. Over the past few decades, there were more and more studies on the treatment of bladder cancer with Chinese herbal monomers. In addition, more and more scholars and professors have made great efforts and published many papers so far, but there is a lack of summary comments. Research in such studies must contribute to obtaining data or information on the latest state of research, measuring current practice, and identifying gaps [10]. Therefore, we believe that it is necessary to make a comprehensive review of this field in order to benefit both new and old researchers in this research field.

Although the traditional literature review is a time-consuming and labor-consuming manual task, which can only provide sampling insights into a certain research field, researchers can conduct bibliometric analysis on a certain research field in order to obtain quantitative and profound knowledge in a relatively short time [11]. Bibliometrics is a convenient new method for qualitative and quantitative analysis of publications [12]. The knowledge graph drawing tools represented by VOSviewer and CiteSpace, through the organic combination of multidisciplinary methods such as applied mathematics, statistics, bibliometrics, and informatics, intuitively show the development process and research hotspot of a research field [13]. Using this method, researchers can quickly and deeply study the theme evolution, main research fields, and new research directions in a certain research field [14]. Over the years, bibliometric analysis has been applied to aristolochic acid [15], health-related quality of life [16], tuberculosis [10], podocyte [12], butyrophilins [17], macrophages associated with acute lung injury [18], and other research areas. However, bibliometric studies on the treatment of bladder cancer with Chinese herbal monomers are still blank. Therefore, the purpose of this study is to systematically analyze the research of Chinese herbal monomer in the treatment of bladder cancer, in order to evaluate the research status and hotspot in this field.

## 2. Materials and Methods

### 2.1. Data Sources and Search Strategies

**Figure 1:**
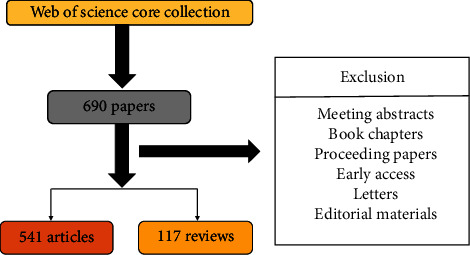
Flowchart of the screening process.

To avoid deviations, publication retrieval was conducted on a single day (February 6, 2022) based on Web of Science Core Collection (WoSCC). The publication period in this study was set between 2000 and 2021. The search terms were presented as follows: (TS=(Bladder Neoplasms) OR TS=(Bladder Neoplasia) OR TS=(Bladder Neoplasm) OR TS=(Bladder Tumor) OR TS=(bladder carcinoma) OR TS=(Bladder Cancer) OR TS=(Bladder Malignancy) OR TS=(Bladder Malignant Neoplasm) OR TS=(Bladder Neoplasm, Malignant) OR TS=(Bladder Benign Neoplasms) OR TS=(Bladder Benign Neoplasm)) AND (TS=(Flavonoids) OR TS=(alkaloids) OR TS=(saccharides) OR TS=(Quinones) OR TS=(Phenylpropanoids) OR TS=(Coumarins) OR TS=(Lignans) OR TS=(Terpenoids) OR TS=(volatile oils) OR TS=(Triterpenes) OR TS=(Steroids) OR TS=(glycosides)). The unstable nature of tannin makes the traditional Chinese medicine preparation easy to precipitate and change color and turbidity, thus affecting the quality of the preparation. Therefore, tannin is regarded as an impurity in many traditional Chinese medicines; therefore, tannin was excluded from the search term of this study. Only original articles and reviews written in English were included among various publication types. In total, 658 articles were ultimately analyzed in our research. The detailed screening is shown in Figure 1.

### 2.2. Data Collection and Cleaning

First, raw data is extracted from WoSCC. Information recorded includes number of papers and citations, *H*-index, year of publication, country/region, institution, author, journal, references, and keywords. Although inaccurate analysis may not be completely avoided due to the multiple editions of the cited literature, the same acronyms of different authors, and the different forms of the cited journals, we believe that most of the original data are reliable. Prior to data analysis by VOSviewer v.1.6.15.0 (Research Centre for Science and Technology, Leiden University, Leiden, Netherlands), CiteSpace was used to combine several duplicates into a single word, correct misspelled elements, and remove useless words. Finally, the cleaned data was imported into VOSviewer and CiteSpace for bibliometric analysis.

### 2.3. Bibliometric Analysis

In this study, VOSviewer (version 1.6.10) and CiteSpace (version 5.8.R3) were used for bibliometric analysis. VOSviewer [19] is used to visualize complex cocitation networks, such as cooperation and time trends among countries, institutions, and individuals. The size of the node represents the number of publications; the thickness of the line represents the strength of the connection; the colors of the nodes represent different clusters or times. CiteSpace is used to facilitate visual analysis of knowledge domains and emerging trends, including cluster analysis, timelines, and keyword bursts. In addition, we analyzed the number of publications (Np), the number of unself-cited citations (Nc), and the *H*-indices of authors, countries, journals, and institutions. In addition, the impact factor (IF) obtained from the latest edition of the Journal Citation Report (JCR) is regarded as one of the main indicators to measure the quality and impact of medical journals [20].

## 3. Results

### 3.1. An Overview of Publications on Treatment of Bladder Cancer with Chinese Medicine Monomer

According to the search strategy, a total of 658 articles and reviews published in the past 22 years were retrieved. The total Nc of the retrieved articles was 18,564, and the average Nc of each article was 29.16. The *H*-index for all publications is 69.

### 3.2. The Annual Trend of Paper Publication Quantity

**Figure 2:**
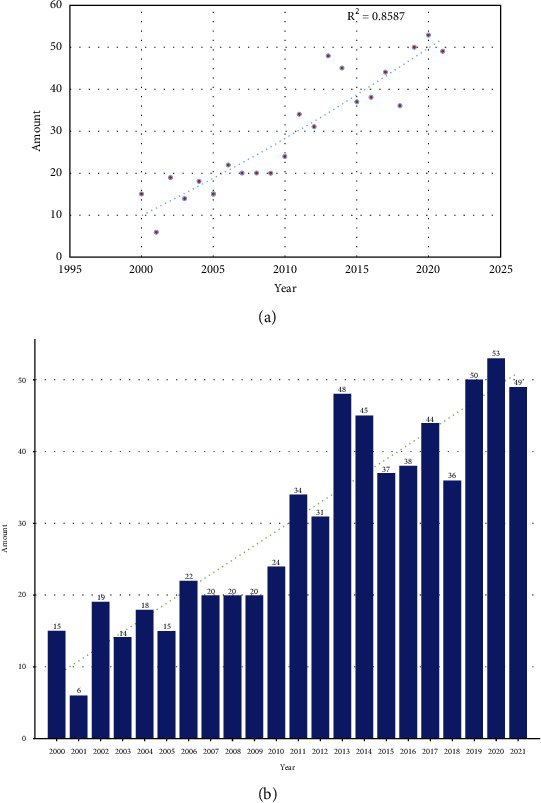
(a) Curve fitting of the of the total annual growth trend of publications (*R*^2^ = 0.8568). The number of publications by year over the past 22 years.

**Figure 3:**
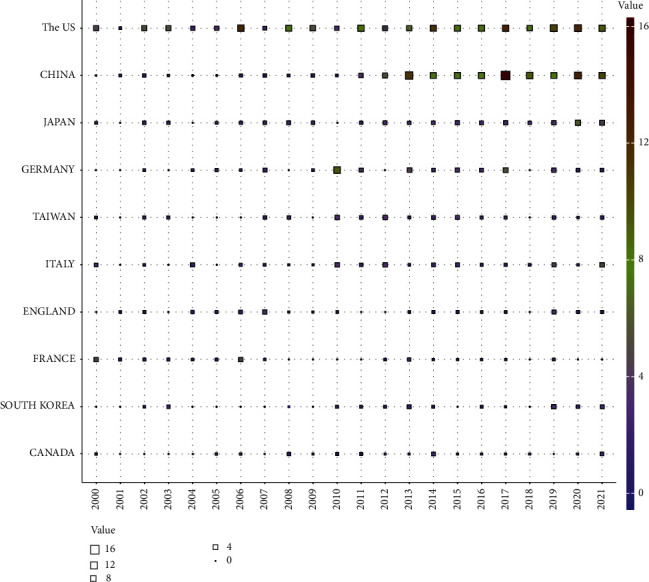
Annual contributions according to country.

Figure 2(a) shows a polynomial fitting curve of the annual trend in the number of printed publications. The annual Np was not significantly correlated with the year of publication, and the correlation coefficient *R*^2^ reached 0.8568 according to Figure 2(a).  Figure 2(b) shows the annual Np related to treatment on bladder cancer with Chinese medicine monomer. Despite fluctuations over the 22-year period, the number of annual papers increased from 177 in 2011 to 413 in 2020, with Np peaking in 2020. Since 2011, the annual Np of the United States and Japan has remained stable, while that of China has increased rapidly. Overall, these findings indicate that research on treatment on bladder cancer with Chinese medicine monomer is still a lot of room for development. On the whole, there is a small amount of literature, and further research is needed.  Figure 3 shows the top ten countries in the number of documents issued. The number of documents issued by each country fluctuated in these 22 years.

### 3.3. Contributions of Countries/Regions to Global Publications

**Figure 4:**
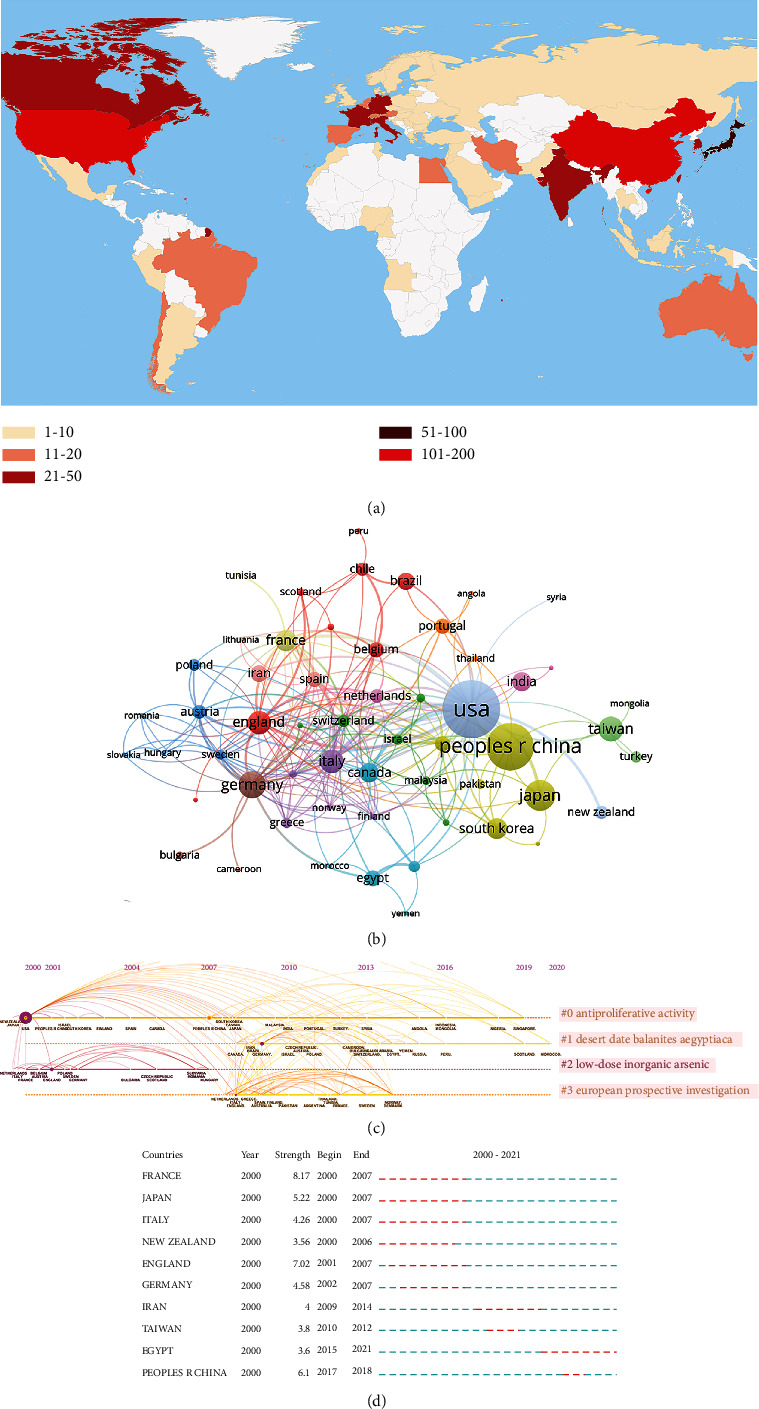
Visualization of country/region analysis. (a) Geographical distribution of global output; (b) visual cluster analysis of cooperation among countries; (c) timeline distribution of cluster analysis of country; and (d) top 10 representative countries burst with the strongest citation bursts.

We ranked the 10 high-producing countries/regions by Np for all authors (Table 1). The United States published the most articles (165), followed by China (115) and Japan (54). Papers from the United States were cited 7,287 times, accounting for 39.25 percent of total citations, followed by China (2,197) and Canada (1945). In addition, the United States had the highest H-index (44), more than twice that of any other country except China. Although the Np of Canada is the lowest, the Nc is higher than other countries except the United States and China and has the highest average per item, indicating that the quality of papers published by Canada in this field is very high.  Figure 4(a) shows the geographical distribution of global output.  Figure 4(b) shows the network of country cooccurrence. The United States and China have the largest number of posts, indicating the highest number of publications. Furthermore, we performed a visualized timeline for clusters, and there were four clusters including antiproliferative activity, desert date Balanites aegyptiaca, low-dose inorganic arsenic, and European prospective investigation.  Figure 4(d) shows the most representative countries in terms of burst strength, burst duration, and burst time. Egypt has the highest burst in recent years.

### 3.4. Analysis of Affiliations and Authors

**Figure 5:**
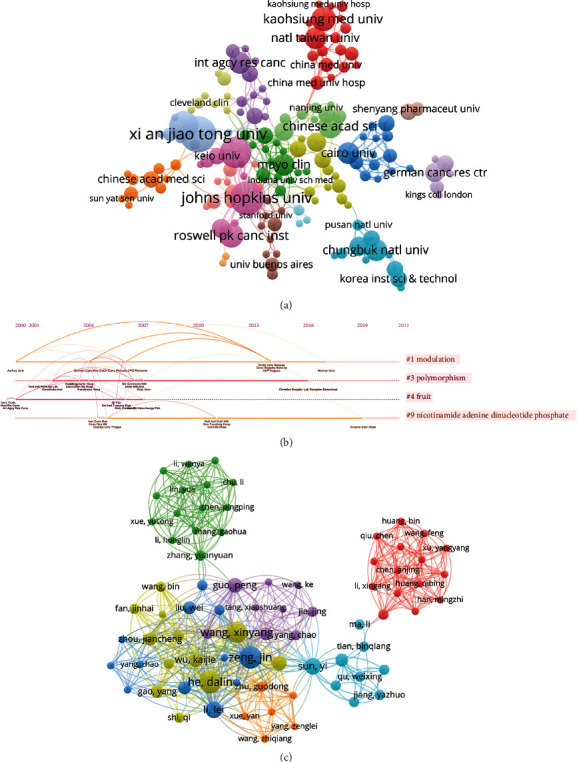
Visualization of affiliation and author analysis. (a) Visual cluster analysis of cooperation among affiliations; (b) timeline distribution of cluster analysis of affiliation; and (c) visual cluster analysis of cooperation among authors.

Table 2 shows the top 10 affiliates with the largest number of publications related to TCM monomer therapy of bladder cancer. Johns Hopkins University has the highest Np [17], followed by the Egyptian Knowledge Base (EKB) [16] and the University of Texas System [15]. The University of Texas System ranked first in Nc (1097), and Jilin University had the highest *H*-index. Although the Egyptian Arab Republic KB EKB has a relatively high Np, its *H*-index lags far behind the University of Texas System, University, Porto, University of California System, and AGREST New Zealand.  Figure 5(a) shows the cooccurrence of the relational network. Among its Chinese affiliates, Xi'an Jiaotong University performs well.  Figure 5(b) shows the subordinate clusters, the first four of which are modulation, polymorphism, fruit, and nicotinamide adenine dinucleotide phosphate.  Table 3 lists the top 10 prolific authors. He published 94 papers, accounting for 14.29% of the total. Miyamoto of the University of Rochester ranked first in the research field of TCM monotherapy for bladder cancer, followed by Munday, CM and Munday, R of Ruakura Agr Res Ctr of New Zealand. As shown in Table 3, Nc of Munday, CM and Munday, Rhad is very high.  Figure 5(c) shows the simultaneous occurrence of the authors.

### 3.5. Analysis of Journals

As shown in Table 4, BJU International (9 publications, IF: 5.588) published the most papers on TCM monomer therapy of bladder cancer, followed by carcinogenic effects (9 publications, IF: 4.944) and molecules (8 publications, IF: 4.412). 11.25% were published in the top 10 academic journals (74/11.25%). Of the top 10 journals, all but the International Journal of Nutrition and Cancer (IF: 2.9) and Cancer Research (IF: 2.48) had a high IF (defined as greater than 3000). It is noteworthy that the International Journal of Molecular Science (IF = 5.924) has a lower citation rate and *H*-index. However, the impact factors of the top ten journals are lower than 10, which indicate that the depth of research in this field needs to be strengthened.

### 3.6. Analysis of Cocitation

**Figure 6:**
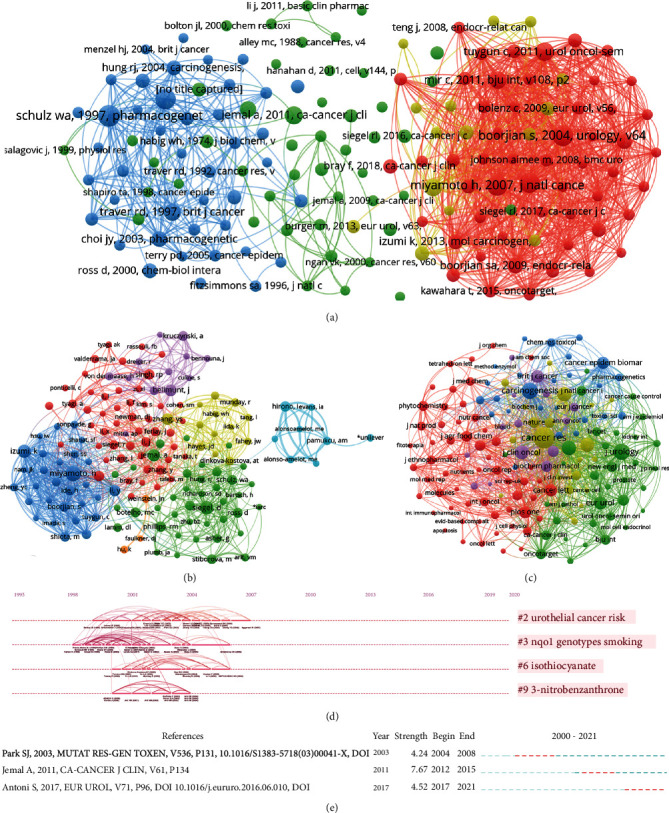
Cocitation analysis. (a) Cocitation analysis of documents; (b) cocitation analysis of authors; and (c) cocitation analysis of journals.

Cocitation refers to when two authors' academic papers are cited by the same paper at the same time. Being able to form a cocitation shows that the concepts, methods, and theories of the two authors for a certain research are related. Therefore, mining the cocitation relationship between authors is helpful to find authors with similar research directions, so as to form a collection of similar scholars. Cocitation analysis can also be used for magazine and literature analysis. The analysis of cocitation is shown in Figure 6. The top ten cocited authors were Miyamoto, H (77 times), Siegel, D 58 (times), Izumi, K 57 (times), Jemal, A (48 (times), Bellmunt, J (47 times), Zhang, YS (44 times), Li, Y 2 (times), Hirono, I (36 times), Phillips, RM (36 times), and Singh, RP (36 times). The top ten cocited references were Schulz, WA (1997, pharmacogenetics, v7, p235, 10.1097/00008571-199706000-00008, 32 times) Boorjian S (2004, urology, v64, p383, 10.1016/j.urology.2004.03.025, 30 times), Miyamoto, H (2007, j natl cancer i, v99, p558, 10.1093/jnci/djk113, 30 times), Miyamoto, H (2012, bju int, v109, p1716, 10.1111/j.1464-410x.2011.10706.x,28 times), Park, SJ (2003, mutat res-gen tox en, v536, p131, 10.1016/s1383-5718(03)00041-x, 28 times), Tuygun, C (2011, urol oncol-semin ori, v29, p43, 10.1016/j.urolonc.2009.01.033, 23 times), Jemal, A (2011, ca-cancer j clin, v61, p134, [10.3322/caac.2011510.3322/caac.20107], 22 times), Mir, C (2011, bju int, v108, p24, 10.1111/j.1464-410x.2010.09834.x, 21 times), Shen, SS (2006, cancer-am cancer soc, v106, p2610, 10.1002/cncr.21945, 21 times), and Traver, RD (1997, brit j cancer, v75, p69, 10.1038/bjc.1997.11, 20 times). The top ten cocited journals were cancer res (865 times), carcinogenesis (516 times), j urology 506 (times), int j cancer (402 times), j biol chem (395 times), p natl acad sci usa (394 times), j clin oncol (367 times), eur urol 329 (times), brit j cancer (317 times), and cancer lett (292 times), Furthermore, we performed a visualized timeline for clusters (Figure 6(d)). We found that “urothelial cancer risk,” “nqo1 genotypes smokings,” “isothiocynante,” and “3-nitrobenzanthrone” are early fields. Finally, we conducted a reference burst. We found that the works of Park, SJ

### 3.7. Analysis of Bibliographic Coupling

**Figure 7:**
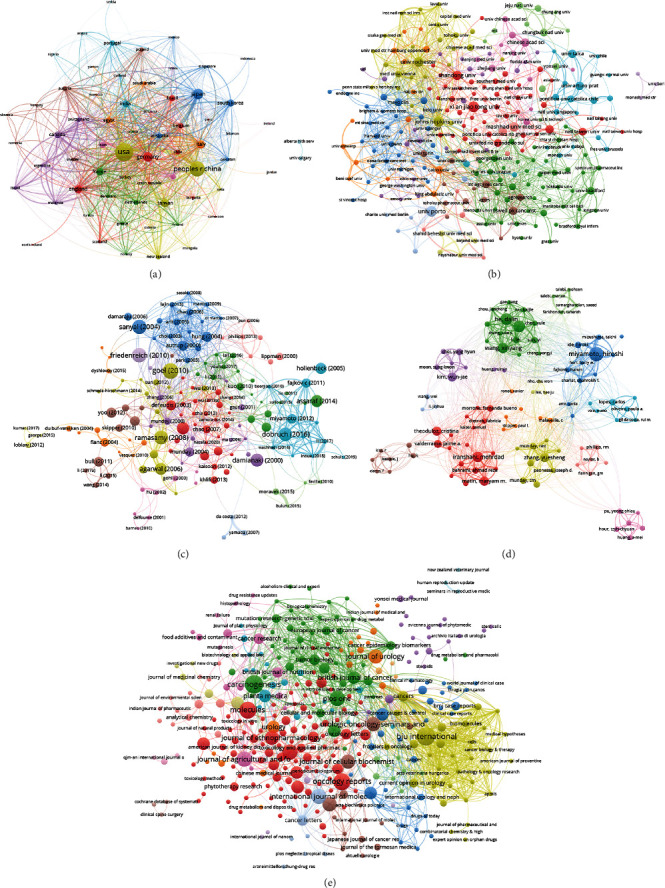
Bibliographic coupling analysis. (a) Network on countries; (b) network on affiliations; (c) network on documents; (d) network on authors; and (e) network on journals.

If two documents cite the same reference, they are said to have a coupling relationship. The number of references cited by two documents is the coupling strength, which reflects the structural relationship and closeness of the relationship between the documents. Analysis of bibliographic coupling was shown in Figure 7. The top ten countries in bibliographic coupling analysis were the US (7372 times), China (2250 times), Canada (1954 times), Germany (1610 times), France (1498 times), Japan (1091 times), Austria (1025 times), Italy (1023 times), England (1009 times), and Portugal (895 times). The top ten affiliations in bibliographic coupling analysis were German Canc Res Ctr (649 times), Karolinska Inst (648 times), Univ Minho (648 times), Univ Colorado (614 times), Columbia Univ (576 times),Univ Buenos Aires (532 times), Univ Sains Malaysia (516 times), Johns Hopkins Univ (509 times), Tel Aviv Univ (493 times), and Univ Alberta (422 times). The top ten documents in bibliographic coupling analysis were Russell (2006, 612 times), Pandi-Perumal (2008, 477 times), Goel (2010, 326 times), Friedenreich (2010, 286 times), Sanyal (2004, 280 times), Ramasamy (2008, 271 times), Dobruch (2016, 261 times), Damianaki (2000, 244 times), Donnelly (2008, 227 times), and Nakamura (2008, 222 times). The top ten journals in bibliographic coupling analysis were the Carcinogenesis (910 times), Phytochemistry (662 times), Nutrition and Cancer-An International Iournal (549 times), Progress in Neurobiology (477 times), Cancer Letters (422 times), BJU International (365 times), European Journal of Cancer (345 times), Journal of Agricultural and Food Chemistry (340 times), Journal of Cellular Biochemistry (308 times), and Journal of Urology(300 times). The top ten authors in bibliographic coupling analysis were Shariat, Shahrokh F. (480 times), Zhang, Yuesheng (356 times), Miyamoto, Hiroshi (314 times), Boorjian, Stephen A. (308 times), He, Dalin (267 times), Malaveille, C (265 times), Munday, CM (251 times), Munday, R (251 times), Zeng, Jin (248 times), and Fajkovic, Harun (241 times).

### 3.8. Analysis of Research Hotspots

**Figure 8:**
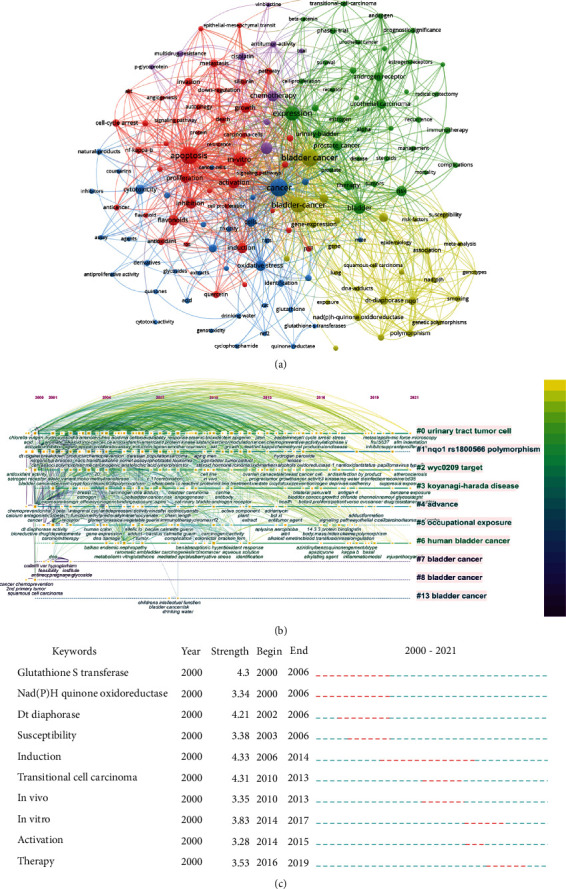
Visualization of keyword analysis. (a) Network keyword cooccurrence; (b) timeline distribution of cluster analysis of keyword; and (c) the top 10 representative burst keywords.

Apart from search terms, keywords extracted from the titles and abstracts of 658 papers were analyzed by VOSviewer and CiteSpace (Figure 8). According to Figure 8(a), clusters 1 and 5 were mainly about the research focuses on the efficacy of monomer compounds in the treatment of bladder cancer; the most involved compounds are flavonoids. Cluster 2 mainly reflected therapeutic effects of steroids on bladder cancer. Cluster 3 focused on cell and animal experiments. In addition to exploring the therapeutic mechanism of compounds for bladder cancer, the toxicological aspects of compounds are also described. Cluster 4 was mainly about applications of modern biotechnology, such as genomics, metabonomics, and epigenetics in bladder cancer. The top frequent occurrences of keywords were “cancer,” “apoptosis,” “bladder cancer,” “expression,” “in-vitro,” “carcinoma,” and “activation,” suggesting that the researches related to treatment on bladder cancer with Chinese medicine monomer mainly focused on basic studies. As shown in Figure 8(b), the timeline of clustering showed that “urinary tract tumor cell” and “wyc 0209 target” were the most important areas of treatment on bladder cancer with Chinese medicine monomer. As shown in Figure 8(c), we found that “glutathione S-transferase” and “nad(p)h quinone oxidoreductase” had the highest burst strength. In addition, we also found that “therapy” and “in vitro” were the keywords that emerged in the last 5 years.

## 4. Discussion

In this study, VOSviewer and CiteSpace software were used to make a bibliometric analysis of the research trends and hotspots in the treatment of bladder cancer with TCM monomaterial in WoSCC database. We searched 658 original articles and reviews published between 2000 and 2021. According to the polynomial fitting curve, the number of annual publications generally showed an upward trend, rising rapidly in the second half of the period, especially after 2013. Among the top countries/regions, the United States ranked first in Np, indicating that the United States was the country with the highest production in this field, the possible reason is that the United States' scientific and technological strength, economic strength, innovation ability, investment, and research depth in this field are much higher than those of other countries. Four American branches and three Chinese authors were among the top 10, which means the US has the world's top research institutions and China has the world's most professional researchers, which partly explains why China and the US have made rapid progress in this field in the past 22 years. Canada, however, had the highest average per item. This shows that Canadian scholars' research in this field is of high quality and more in-depth than that of other countries in the world. This suggests that scholars and affiliated institutions in other countries should make more efforts to improve the quality of papers in this field. Similarly, there is a contradiction between quantity and quality of publications in France.

Notably, seven of the 10 most productive journals had higher IF. This means that it is not a challenge to publish a study on the treatment of bladder cancer with Chinese herbal monomers.

With the deepening of research, people have found signaling pathways involved in various mechanisms. Keyword mapping revealed that nuclear transcription factor (NF-*κ*B) plays a key role in the pathogenesis of bladder cancer. Nuclear factor *κ*B (NF-*κ*B) has been identified as a regulator of the *κ*B light chain in mature B cells and plasma cells [21]. NF-*κ*B plays an important role in the development and progression of cancer [22]. Bladder cancer is the second most common cancer of the urinary system, and NF-*κ*B involvement in the development of superficial or muscle infiltrating disease and cancer recurrence is preliminary at best [23]. Constitutive NF-*κ*B activation was observed in high-grade bladder cancer [24, 25]. Geng et al.'s research suggest that the NF-*κ*B pathway regulates the expression of bladder EMT and CSC markers induced by tobacco smoke, as well as the protective effect of tobacco smoke that NF-*κ*B pathway regulates the expression of bladder EMT and CSC markers and the protective effect of DATS induced by tobacco smoke [26]. Multiple molecules could enhance bladder cancer cell migration, invasion, and proliferation by activating NF-*κ*B signaling [27, 28]. Some compounds can also inhibit bladder cancer by inhibiting NF-kappa B [25, 26, 29]. This study shows that quercetin is the most studied monomer compound in all traditional Chinese medicine. Quercetin is a natural flavonoid from plants. It has good therapeutic effect on many diseases and has good anti-inflammatory and antioxidant effects [30], which is a widely distributed plant secondary metabolite. Quercetin widely exists in daily diet and nature. It can produce toxicity to cancer cells without damaging healthy cells and has therapeutic effects on a variety of cancers. Quercetin is lipophilic and can smoothly cross the cell membrane and play an antitumor role. Its main mechanism is to block the invasion and metastasis of cancer cells, induce apoptosis of cancer cells, and regulate and inhibit the expression of oncogenes. The study of Adami et al. demonstrated that quercetin had antitumor effect on human bladder cancer cells, and the activity and proliferation of T24 cells decreased. Biophysical and morphological analyses showed that this effect was the result of quercetin-induced apoptosis, as the treated T24 cells showed morphological changes characteristic of apoptosis. In addition, quercetin-treated T24 cells showed no plasma membrane damage, suggesting that cell death was not due to necrosis but to apoptosis. Further NMA analysis showed that T24 cells showed fewer normal nuclei and more nuclear changes common during early apoptosis, senescence, and other nuclear damage, thus confirming the apoptotic effect of quercetin. Finally, AFM analysis revealed a pattern of cell damage after treatment with different quercetin concentrations, gradually beginning with irregular small contours, increasing the number of holes and pits and cell shrinkage, and eventually causing tumor cell collapse. Finally, our study shows for the first time the morphological and mechanical characteristics of quercetin-treated bladder cancer T24 cells and suggests that it has antitumor effects. We hope this study will serve as a starting point and support for other studies to analyze quercetin for bladder cancer [31]. Ma et al. demonstrated that quercetin inhibits the growth and proliferation of bladder cancer cells and induces apoptosis, which is associated with reduced DNA methylation levels of P16, RASSF1A, and Er*β* genes and reduced expression of mutP53 and Survivin proteins. Quercetin is a potential chemoprophylaxis and chemotherapy agent for bladder cancer. Further studies of demethylation genes and downstream molecular mechanisms associated with the cell cycle are needed [32]. The study of Orsolic showed that quercetin can inhibit the proliferation and colony formation of human bladder cancer cells by inducing DNA damage, and quercetin may be an effective chemoprophylaxis and chemotherapeutic agent for papillary uroepithelial bladder cancer after transurethral resection [33]. All these suggest that quercetin may be the most promising monomer for the development of bladder cancer drugs. Another well-studied compound is silibinin. Silibinin is a silymarin extracted from the fruit of silymarin, which is a mixture of active flavonoid lignin and flavonoids [34], which has many physiological functions, such as protecting myocardium, reducing blood lipid, protecting liver, scavenging free radicals, and antioxidation. [35] Silibinin can induce apoptosis of human bladder cancer cell line T24 and 5637, thus inhibiting cell invasion and proliferation [36]. Silibinin can reduce the proliferation and increase the apoptosis of TP53 mutant cells. In both RT4 and T24 cell lines, increased early apoptosis rate, primary DNA damage, and reduced cell colonies in clonal survival tests were detected. Expression of FRAP/mTOR, AKT2, FGFR3, DNMT1, and miR100 were downregulated in RT4 cells. Regulation of miR203 was observed in both cell lines [37].

## 5. Conclusion

This analysis of bibliometric reveals that researches on treatment on bladder cancer with Chinese medicine monomer is still much room for development, and the current research literature is less at present. The United States is a major producer and has made many outstanding breakthroughs in this field. The BJU International and Carcinogenesis published the latest research and new advances in this field. In recent years, the role of NF-*κ*B molecular pathway has become a focus of research. Moreover, quercetin has been studied most, and it may be a promising new drug in treating bladder cancer.

## Figures and Tables

**Table 1:** Publications in the 10 most productive countries/regions.

Rank	Country/region	NP	NC	*H*-index	Average per item
1	The US	165	7287	44	44.75
2	China	115	2197	24	19.57
3	Japan	54	1083	20	20.2
4	Germany	44	1596	20	36.59
5	China Taiwan	37	747	16	20.38
6	Italy	34	1013	17	30.09
7	England	33	985	17	30.58
8	France	28	1489	18	53.5
9	South Korea	27	654	13	24.37
10	Canada	24	1945	17	81.42

**Table 2:** The top 10 productive affiliations.

Rank	Affiliation	Np	Nc	Country	*H*-index	Average per item
1	Johns Hopkins University	17	897	The US	13	53.29
2	Egyptian Knowledge Bank EKB	16	207	The Arab Republic of Egypt	8	12.94
3	University of Texas System	15	1097	The US	10	73.33
4	Universidade do Porto	13	226	The Portuguese Republic	9	18.38
5	University of California System	13	582	The US	10	45.08
6	Xi'an Jiaotong University	13	325	China	10	26.23
7	University of Rochester	11	297	the US	6	28.73
8	AgResearch New Zealand	10	450	New Zealand	9	46.7
9	Mashhad University Medical Science	10	138	Iran	8	15.7
10	Shandong University	10	232	China	7	23.2

**Table 3:** The top 10 authors with the most publications.

Rank	Author	Np	Affiliation	Country	Nc	*H*-index	Average per item
1	Miyamoto H	11	Univ Rochester	USA	292	6	28.55
2	Munday CM	10	Ruakura Agr Res Ctr	New Zealand	450	9	46.7
3	Munday R	10	Ruakura Agr Res Ctr	New Zealand	450	9	46.7
4	Wang XY	10	Minist Educ	China	248	8	26
5	Bahrami AR	9	Ferdowsi Univ Mashhad	Iran	117	7	15.11
6	He DL	9	Xi An Jiao Tong Univ	China	255	7	29.67
7	Iranshahi M	9	Mashhad Univ Med Sci	Iran	117	7	15.11
8	Matin MM	9	Ferdowsi Univ Mashhad	Iran	117	7	15.11
9	Zeng J	9	Xi An Jiao Tong Univ	China	235	7	27.56
10	Phillips RM	8	Univ Bradford	England	186	7	18.45

**Table 4:** The top 10 most active journals.

Rank	Journal	Np	IF (2020)	Nc	*H*-index	Average per item
1	BJU International	9	5.588	365	9	40.56
2	Carcinogenesis	9	4.944	909	9	101.11
3	Molecules	8	4.412	130	8	16.5
4	Oncology Reports	8	3.906	130	7	16.38
5	International Journal of Molecular Sciences	7	5.924	161	4	23
6	Journal of Agricultural and Food Chemistry	7	5.279	338	7	48.57
7	Nutrition and Cancer-An International Journal	7	2.9	548	6	78.43
8	PLOS One	7	3.24	250	6	35.71
9	Anticancer Research	6	2.48	291	5	48.5
10	Journal of Cellular Biochemistry	6	4.429	308	6	51.33

## Data Availability

The data could be acquired from the corresponding author.
